# Microarchitecture of titanium cylinders obtained by additive manufacturing does not influence osseointegration in the sheep

**DOI:** 10.1093/rb/rbab021

**Published:** 2021-06-25

**Authors:** Louis Rony, Eric Aguado, Bruno Verlee, Florence Pascaretti-Grizon, Daniel Chappard

**Affiliations:** GEROM—Groupe Etudes Remodelage Osseux et bioMatériaux, LabCom NextBone, Univ-Angers, IRIS-IBS Institut de Biologie en Santé, 49933 Angers, France; GEROM—Groupe Etudes Remodelage Osseux et bioMatériaux, LabCom NextBone, Univ-Angers, IRIS-IBS Institut de Biologie en Santé, 49933 Angers, France; ONIRIS, Ecole Vétérinaire de Nantes, 44307, Nantes Cedex 3, France; SIRRIS Liège Science Park, Rue du bois St Jean 12, Seraing 4102, Belgium; GEROM—Groupe Etudes Remodelage Osseux et bioMatériaux, LabCom NextBone, Univ-Angers, IRIS-IBS Institut de Biologie en Santé, 49933 Angers, France; GEROM—Groupe Etudes Remodelage Osseux et bioMatériaux, LabCom NextBone, Univ-Angers, IRIS-IBS Institut de Biologie en Santé, 49933 Angers, France; SCIAM, Service Commun d'Imagerie et Analyses Microscopiques, IRIS-IBS Institut de Biologie en Santé, Univ-Angers, Angers Cedex 49933, France

**Keywords:** titanium, trabecular scaffold, osseointegration, geometric architecture, histomorphometry

## Abstract

Large bone defects are a challenge for orthopedic surgery. Natural (bone grafts) and synthetic biomaterials have been proposed but several problems arise such as biomechanical resistance or viral/bacterial safety. The use of metallic foams could be a solution to improve mechanical resistance and promote osseointegration of large porous metal devices. Titanium cylinders have been prepared by additive manufacturing (3D printing/rapid prototyping) with a geometric or trabecular microarchitecture. They were implanted in the femoral condyles of aged ewes; the animals were left in stabling for 90 and 270 days. A double calcein labeling was done before sacrifice; bones were analyzed by histomorphometry. Neither bone volume, bone/titanium interface nor mineralization rate were influenced by the cylinder’s microarchitecture; the morphometric parameters did not significantly increase over time. Bone anchoring occurred on the margins of the cylinders and some trabeculae extended in the core of the cylinders but the amount of bone inside the cylinders remained low. The rigid titanium cylinders preserved bone cells from strains in the core of the cylinders. Additive manufacturing is an interesting tool to prepare 3D metallic scaffolds, but microarchitecture does not seem as crucial as expected and anchoring seems limited to the first millimeters of the graft.

## Introduction

Cavitary bone loss is common in orthopedic and traumatology surgery: osteolysis due to wear of polyethylene particles of a total hip prosthesis, comminuted metaphyseal fracture, revision prosthesis with intraoperative fracture. The subsequent bone reconstruction becomes a challenge [1]. For reconstruction of trabecular bone loss, it is necessary to fill the areas with osteoconductive biomaterials to prevent the development of fibrosis and loss of bone tissue function [2, 3].

The biomaterials that can be used are mainly calcium orthophosphate ceramics that do not have stress resistant properties and only allow osteoconduction from the surrounding bone. Phosphocalcic ceramics allow bone regeneration in the injured area as shown in a large number of publications [4–6]. This is possible in maxillofacial surgery but cannot be achieved in orthopedic surgery [7]. Indeed, their lack of mechanical resistance to crushing would require immediate post-operative unloading of the operated area. Moreover, the lack of mechanical stress is also detrimental to the regeneration of the bone tissue [8, 9].

To overcome these disadvantages, certain materials have been developed to counteract the problem of mechanical strength and allow the area of bone loss to be filled while providing mechanical resistance [1, 10–12]. Titanium alloys are commonly used in orthopedic surgery and bone adapts particularly well to titanium, so much so that the word ‘osseointegration’ has been widely used in dental implantology [8, 13]. Furthermore, osteoblasts have a natural affinity for titanium and for concave-convex surfaces, and bone regeneration is favored on non-geometric surfaces [14, 15]. Therefore, it can be hypothesized that titanium implants that mimic a natural bone architecture would allow for better osseointegration than if a geometric architecture was designed as conventionally [16, 17].

The objective of this work was to determine whether the architecture of the component influences its osseointegration. The implantation time was also studied to determine if it had a significant influence. We therefore prepared two types of titanium cylinders by 3D printing (synonyms: 3D printing, rapid prototyping): one reproducing a geometrical architecture similar to the fusion cages used in spine surgery and the other one reproducing the microarchitecture of an ox bone. The results were compared using the same methodology at 3 and 9 months after an implantation in sheep.

## Material and methods

### Biomaterials

**Figure 1.**
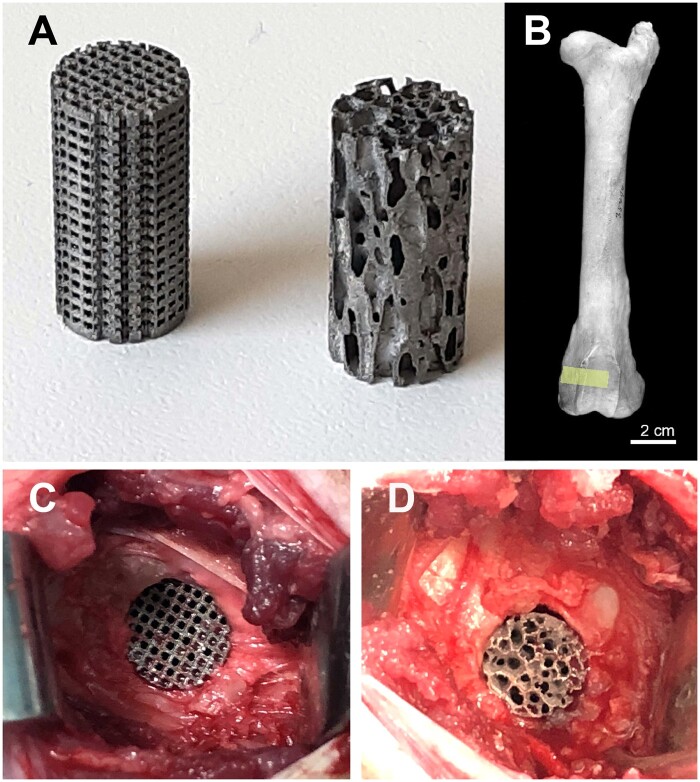
(**A**) Titanium cylinders prepared by additive manufacturing: left: geometric microarchitecture; right: trabecular microarchitecture. (**B**) The surgical site where the implants were placed in the femoral condyle appears in yellow. (**C**) Surgical placement of the two types of implants in the ewe. Right: geometric microarchitecture; left: trabecular microarchitecture

In additive manufacturing, printing with metal powders offers the possibility to produce objects of any shape in a single production step from a 3D model. The 3D model of the cylinders with a trabecular microarchitecture was obtained from the tibial plateau an old ox. This model was chosen after a considerable number of testing because it provided a largest amount of bone, with anisotropic and mature trabecular microarchitecture. In addition, bovine bone has the same microarchitecture [18–20] and biomechanical strength [21] as young humans (human cadavers from anatomy laboratory are most often osteoporotic). The dry sample was analyzed by X-ray microtomography and the images were sent to Sirris to be converted into STL files (Standard Triangle Language). The 3D model of the cylinder with a geometrical microarchitecture was imported into a specific software (e.g. Magics^®^, Materialise Leuven, Belgium) for processing and preparation of the STL file. A support for holding the cylinders was also added to separate them from the built plate of the 3D printer. The STL files combining the cylinders and their supports were then imported into the MTT Autofab Marcam^®^ slicing software (Release 1.6, Renishaw Benelux, Brenda, The Netherlands) to virtually slice the parts into 30-µm thin layers. Contours were produced at 100 W and 500 mm/s, and the internal hatching at 190 W and 600 mm/s, with a hatch distance of 120 µm between passes. The sliced files were sent to the additive printer (SLM 250HL^®^, SLM Solutions, Lübeck, Germany). Printing took place in a closed chamber under protective atmosphere (argon), spreading successive layers of powder and melting them selectively to build up the parts layer by layer. The build plate was heated to 100°C to limit internal stress in the material. The powder material used was an argon atomized Ti6Al4V ELI titanium alloy powder from TLS Technik (Bitterfeld-Wolfen, Germany) with a spherical shape and a particle size dispersion of 20–63 µm. After fabrication, the cylinders were cut out of the tray using a band saw without lubricating liquid. A maximum of unmelted powder was manually removed from the cylinders in successive steps using a vacuum cleaner and a vibrating table. This cleaning step was completed by a passage in an ultrasonic bath under isopropanol. The cylinders were then placed in a drying oven and the supports were removed. The resulting cylinders were 1 cm in diameter and 2 cm in length (Fig. 1A).

### Biomaterial cleaning

Cylinders obtained by additive manufacturing require post-processing to improve surface parameters such as roughness and to remove unmelted metallic powder particles. Removal of these unmelted particles as well as the etching of the surface was achieved by a chemical post-processing using a hydrofluoric/nitric acid (HF/HNO_3_) solution. The method was adapted from those used to clean dental implants [22]. Briefly, the cylinders were treated by strong acids (HNO_3_ 65% (5 vol.) + HF 58% (1 vol.) and cleaning solutions (sodium perborate, tetracetylene diamine and tensio-active reagents). A thorough washing was performed in distilled water using ultrasonic waves during cleaning. The cylinders were dried and sterilized by gamma radiation.

### Scanning electron microscopy

Surface morphology of the raw and cleaned cylinders was analyzed on an EVO LS10 (Carl Zeiss Ltd., Nanterre, France). Images were captured in the secondary electron mode with an acceleration tension of 20 kV.

### Computational fluid dynamics through the cylinders

The cylinders were transferred to an X-ray computed nanotomograph (Nanotom, Phoenix, GE, USA) using polychromatic X-rays to limit artefacts due to titanium. NanoCT analysis was performed at 100 kV and 220 µA and with a copper filter. Five projection images were averaged every 500 ms and the rotation angle was 0.2° with a pixel size of 12.82 µm. Image reconstruction was done on the projection images with DATOS software (Phoenix, GE) and a stack of 2D sections was obtained for each specimen.

Morphometric analysis of the cylinders was performed with CTAn software (Bruker microtomography, Kontich, Belgium) and the following parameters were determined:

- Porosity (expressed in %) represents the overall amount of the cylinder occupied by pores,
- Pore diameter (in µm),
- Thickness of the titanium trabeculae (in µm) and
- Density of titanium trabeculae (in/mm).

The 3D models were obtained with VG StudioMax 3.2 (Volume Graphics GmbH, Heidelberg, Germany). Once the 3D models were obtained, the ‘Transport Phenomena Simulation’ add-on module of VG Studiomax was used to compute computational fluid dynamic (CFD) simulations on the two types of cylinders [23]. The boundary conditions were defined as follows: first, the cylinder was cut to obtain a square-section parallelepiped, aligned with the major axis of the cylinder. A superior plan was defined; then, a opposite parallel plane (separated by 6700 µm) was selected. The following parameters were used for the simulation: an outlet pressure of 1 Pa; then, the wall boundaries were defined as sealed faces, the physical properties for the dynamic viscosity of the fluid was set at η = 0.00155 Pa.s (similar to that of extracellular fluids) [24]. The computation was performed with a simulation cell size of 3-voxel and 1000 iterations. The following parameters were obtained:

- Absolute permeability (k): it is a measure of the capacity of a cylinder to let fluids pass. The unit of measurement is the milliDarcy [25, 26].
- Hydraulic tortuosity (T): it is a parameter describing the average elongation of fluid streamlines in a porous medium relative to a free flow (with a value = 1). Hydraulic tortuosity is a dimensionless quantity, but is always > 1 [27, 28].
- Total flow rate: it indicates the total volume of fluid per second transported through the cylinder volume [29]. The unit of measurement is mm^3^/s.

In addition, the graphical analysis illustrates the calculated velocity distribution in a color-coded manner according to a LUT (look up table). The streamlines, superimposed in white, illustrate the flow direction between the titanium cylinder meshes.

Morphometric and CFD analysis were done in triplicate for both cylinder types.

### Animal and surgical procedure

The animal study was conducted from January 2019 to October 2019 with two parallel open arms for 270 days. Nine ewes sacrificed after 90 days constituted the first group (Group A) and nine ewes sacrificed after 270 days constituted the second group (Group B). The subjects of the study were cull Vendean ewes (non-GMOs (non-genetically modified organisms), ∼6 years old) from local breeders. The ewes were acclimated at the Veterinary School of Nantes’ sheepfold (ONIRIS, France) between 10 and 21 days before surgery. They were operated by a trained veterinarian surgeon in an operating room dedicated to animal experimentation at ONIRIS.

The ewes were operated under general anesthesia performed with a standardized protocol including two hypnotic drugs by intravenous infusion (Isoflurane and Ketamine) and a benzodiazepine (Diazepam). General anesthesia was maintained after endotracheal intubation with halothane supplied by an anesthetic machine. An injection of amoxicillin trihydrate (500 mg) was administered before the incision. The field allowed access to the posterior and the medial side of both knees. A skin incision was made longitudinally, parallel to the axis of the femur. The subcutaneous tissues were carefully coagulated to avoid postoperative hematoma. The medial side of medial femoral condyle was reached through the *sartorius* muscle. The periosteum was carefully dissected and a blind tunnel (1 cm in diameter, 2 cm in depth) was made using progressively sized drills with a low speed (400 rpm) electric rotary instrument under physiologic saline irrigation to remove bone debris (Fig. 1B). Titanium cylinder was impacted in these blind tunnels; left knees were filled with a geometric cylinder and right knees with a trabecular cylinder. Once the tunnel was filled, the periosteum was carefully closed to prevent the graft from spreading into the adjacent soft tissues. The muscle fascia was also sutured with absorbable thread as was the subcutaneous tissue and skin. The dressing was made sterile and stapled before avoiding any contamination in sheepfold.

In Groups A and B, two subgroups were obtained: filling with a geometric titanium cylinder (Subgroup g) or filling with a trabecular titanium cylinder (Subgroup t; Fig. 1C and D). Thus, four subgroups were considered: A-g and A-t subgroups sacrificed at 90 days and Subgroups B-g and B-t sacrificed at 270 days.

Towards the end of each of the two study periods, a double calcein labeling was performed to determine the mineral apposition rate (MAR) and a ‘2 days on, 10 days off and 2 days on’ regimen was used (calcein dose: 30 mg/kg; IM route). Animals were sacrificed by lethal injection of phenobarbital. Both knees were removed for analysis and fixed in 10% formalin. In all groups, an additional bone sample from the tibial metaphysis was harvested for measurement of MAR at distance from the surgical site.

### Histological and histomorphometric analysis

The samples were embedded in poly(methylmethacrylate) for histological analysis as previously described [30]. The blocks were then sectioned with a diamond saw (Accutom, Struers, France) into 500-µm thick slices. The slices passing through the central position of the cylinders were selected. The slices were polished on an automatic polishing machine (Struers) to a 1 µm finish with diamond particles. Then, the slices received one of the following treatments. (i) Slices for fluorescence microscopy were left unstained and analyzed on an Olympus BX 51 microscope (Olympus, France) with a U-MWIB3 cube (excitation filter 460–495 nm, emission filter 510 nm, dichromatic mirror 505 nm). (ii) Surface staining was performed after acid etching of the surface with 0.1% formic acid in distilled water for 4 min followed by staining with borax-toluidine blue during 45 min.

Morphometric analysis was done on a semi-automatic image analyzer. The standardized nomenclature for bone histomorphometry of the ASBMR (American Society for Bone and Mineral Research) was used [31]. The following parameters were determined:

- Volume of titanium in the cylinder volume (TitV/TV, in %),
- Volume of trabecular bone grown inside the cylinder (BV/TV, in %),
- Percent of titanium surface covered by bone (BS/TitS, in %),
- Mineralization rate in bone in direct contact with titanium (MAR_cont_, in µm/D) and
- Mineralization rate in bone at distance (>2000µm) from the cylinder (MAR_dist_, in µm/D).

### Ethical consideration

This project was approved by the National Ethics Committee for Animal Experimentation (CEEA) under the reference number: CEEA.2012.257. This study took into account the 3Rs rule (Replace, Reduce, Refine) aimed at limiting the number of subjects needed for the experimentation [32].

### Statistical analysis

The SYSTAT software (Systat, San José, CA, release no. 13.00.05) was used to perform statistical analysis. All results were expressed as mean ± SD. Due to the small number of bone samples used in this study, a Kruskall–Wallis non-parametric analysis of variance was used to compare differences among groups. The comparison between groups was obtained by *post hoc* tests. Differences were considered significant for *P* < 0.05.

## Results

### Biomaterial scanning electron microscopy

**Figure 2.**
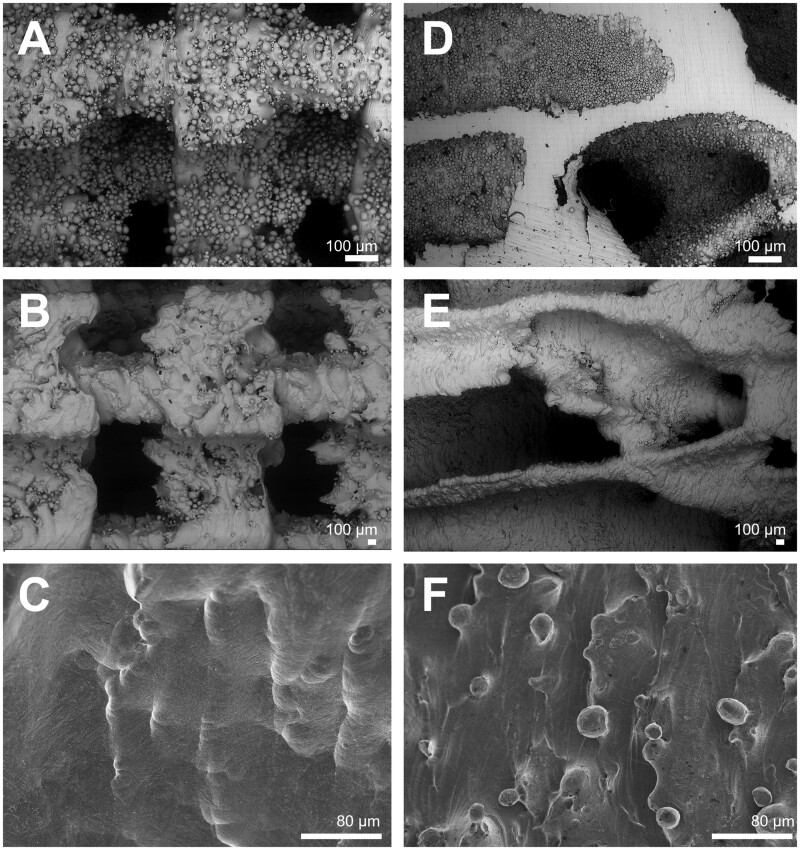
Scanning electron microscopy analysis of the two types of titanium cylinders with a geometric or trabecular microarchitecture. (**A**) Raw cylinder with geometric architecture showing the presence of numerous elementary microbeads. (**B**) Same sample after treatment with HF/HNO_3_, note the disappearance of microbeads and many asperities on the titanium surfaces. (**C**) Same sample at higher magnification: note the smooth surface etched by the chemical treatment. (**D**) Raw cylinder with a trabecular bone architecture showing the presence of numerous elementary microbeads. (**E**) Same cylinder showing the effect of treatment with HF/HNO_3_, microbeads have been removed and many asperities on titanium surfaces. (**F**) Same sample at higher magnification: note the smooth surface etched by the chemical treatment

All cylinders were prepared in the same way; 3D printing provided raw cylinders with imperfections related to the printing technique. In order to avoid the elementary microbeads which that were not fused by the laser used during the 3D printing, from coming off the final component, a HF acid treatment was performed. This treatment resulted in wavy and concave surfaces (Fig. 2).

### Morphometric analysis and CFD through the cylinders

**Figure 3.**
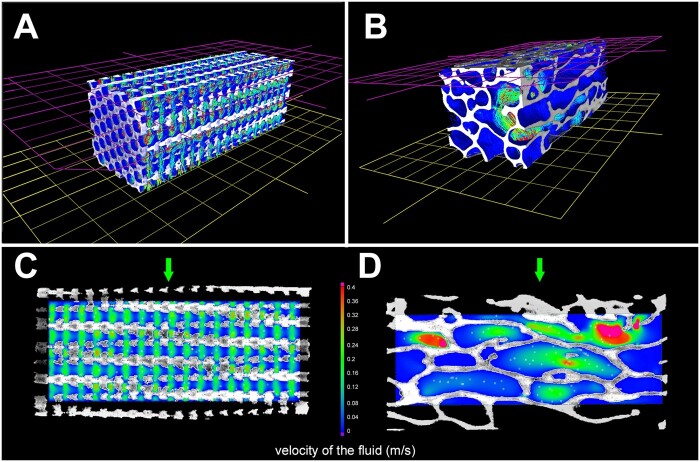
CFD between the two types of cylinders. (**A** and **D**) describe in 3D the principle of the simulated trajectory of a fluid from the pink to the yellow plane. The trajectories of the fluid are illustrated as colored lines. (**C** and **D**) are 2D sections from each type of cylinders showing the flow rate and the direction of the fluid. Velocity is color-coded according to the LUT from 0 to 0.4 m/s; streamlines appear in white. Trabecular cylinders are associated with a very heterogeneous distribution of the flow rate, but the mean flow rate was significantly lower than for geometric cylinders. The direction of the simulated fluid is indicated by a green arrow

The morphometric characteristics of the two types of cylinders are shown on Table 1 with the CFD parameters. The porosity did not differ between the two types of cylinders; but the parameters describing their microarchitecture were significantly different. Figure 3 illustrates the principle and results of the CFD simulation applied to two types of cylinders. Figure 2A and B illustrate the conditions of the simulation applied to the parallelepiped volumes cut into the cylinders: the sidewalls of the volume of interest were cut with a clipping box and defined as no-slip faces. The axis-flow was in the vertical direction starting from the pink inlet plane to the opposite plane in yellow that constituted the outlet face. The calculated fluid velocity is color-coded according to the LUT that was the same for all analyses. The streamlines are also shown. The other pictures correspond to 2 D images of the two types of microarchitecture (geometric or trabecular). It is easy to see that the fluid permeation through the geometric cylinders is rather uniform. On the contrary, the fluid velocity inside the trabecular cylinders is very heterogeneous with areas associated with strong fluid movements and areas with very low flow. The geometric cylinders had significantly higher absolute permeability and flow rate than the corresponding trabecular cylinders. On the contrary, the tortuosity (measuring the length of the flow path) was significantly higher in the trabecular cylinders because the streamlines trajectories were more complex and elongated.

**Table 1.** Morphometric parameters and CFDs through the two types of cylinders

	Unit	Geometric	Trabecular	*P*
Porosity	%	66.7 ± 1.9	68.0 ± 1.28	NS
Pore diameter	µm	981 ± 77	1399 ± 33	0.05
Trabecular thickness	µm	488 ± 10	656 ± 24	0.05
Trabecular density	/mm	0.68 ± 0.03	0.48 ± 0.003	0.05
Absolute permeability	mD (× 10^6^)	4.81 ± 0.8	1.05 ± 0.36	0.05
Tortuosity		1.15 ± 0.09	2.1 ± 0.17	0.05
Flow rate	mm^3^/s	0.38 ± 0.02	0.09 ± 0.03	0.05

### Histological analysis

**Figure 4.**
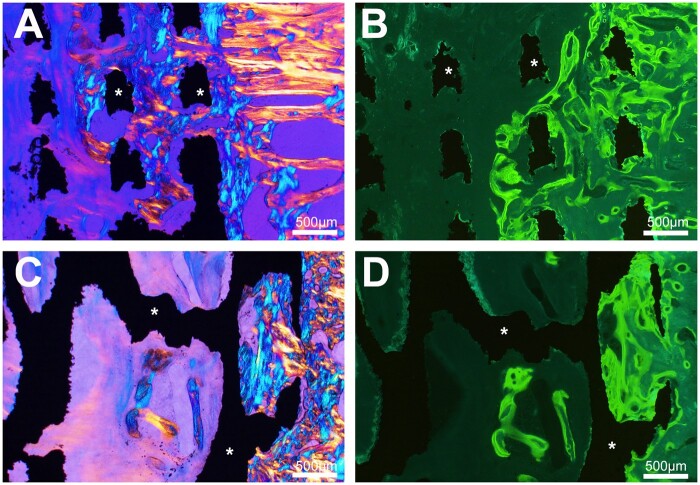
Histological analysis by light microscopy and fluorescence 90 days after cylinder implantation. Osseointegration is noted with both biomaterial whatever its geometry. (**A**) In cylinders with geometrical architecture, trabecular bone extends between the titanium septa (asterisks) from the peripheral zone of anchorage (polarized light). (**B**) fluorescence microscopy of the same cylinder, numerous double labels are evidenced between the biomaterial septa and in the surrounding bone. (**C**) Cylinders with a trabecular architecture, trabecular bone extends from the peripheral area into the cavities limited by the titanium trabeculae (asterisks). (**D**) Fluorescence microscopy of the same cylinder, numerous double labels within are observed in bone within the biomaterial cavities and the surrounding bone

**Figure 5.**
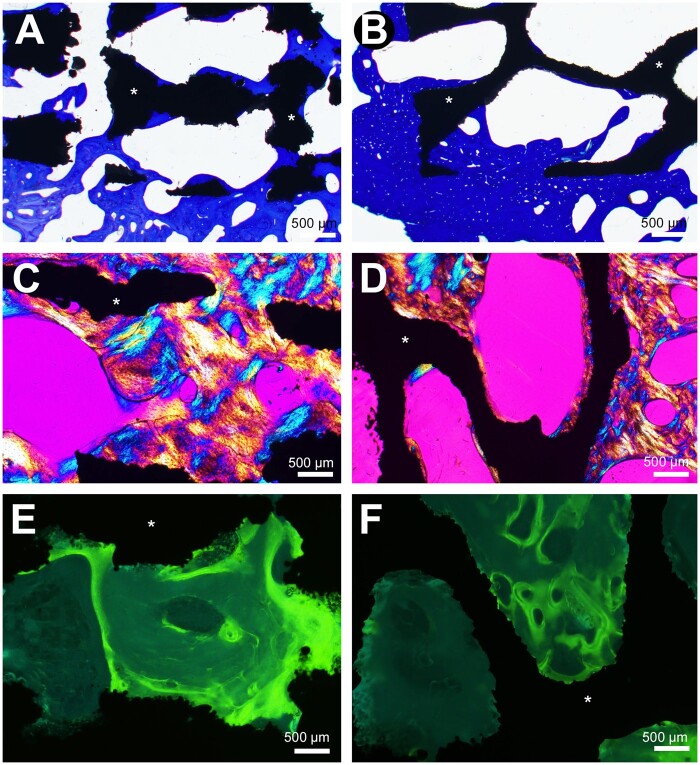
Histological analysis by light microscopy and fluorescence 270 days after cylinder implantation. (**A**) Trabecular bone is in direct contact with titanium septa (asterisks) (toluidine blue). (**B**) Trabecular bone is in direct contact with titanium trabeculae (toluidine blue). (**C**) Trabecular bone creeps between the titanium septa in the geometrical architecture cylinders (polarized light). (**D**) Trabecular bone creeps between the titanium trabeculae (polarized light). (**E**) Fluorescence microscopy, numerous labels are observed in bone within the biomaterial cavities limited by the titanium septa. (**F**) Fluorescence microscopy, numerous labels are observed in bone within the biomaterial cavities limited by the titanium trabeculae

At both 90 and 270 days, all cylinders were osseointegrated with bone anchored to the surface of the material. At 90 days, the presence of trabeculae made of lamellar bone was observed apposed to the titanium. Some trabeculae were also observed within the both types of metal scaffolds, and in polarized light, some areas were composed of woven bone at 90 days but all trabeculae were composed of lamellar bone at 270 days. Under fluorescence microscopy, double labeling was evident on trabeculae located on the outside of the cylinders and anchored to their surface while fuzzy labeling was noted in areas of woven bone. The microarchitecture of the cylinder did not appear to influence osseointegration at 90 days as at 270 days (Figs 4 and 5). Near the cortices, more bone was present extending from the edges of the hole.

### Histomorphometry

Results of histomorphometric parameters are summarized on Table 2.

**Table 2.** Histomorphometric parameters (results are expressed as mean ± SD)

		Group A (90 days)				Group B (270 days)		
	Unit	Subgroup g	Subgroup t	*P*		Subgroup g	Subgroup t	*P*
TitV/TV	%	40.1 ± 4.9	34.1 ± 3.3	0.009		41.6 ± 0.6	36.8 ± 6.2	0.009
BV/TV	%	6.0 ± 3.1	6.6 ± 4.2	NS		9.38 ± 1.8	7.9 ± 3.7	NS
BS/TitS	%	38.2 ± 7.9	23.6 ± 9.8	NS		55.4 ± 9.2	47.3 ± 6.5	NS
MAR_cont_	µm/D	1.97 ± 0.10	2.02 ± 0.16	NS		1.63 ± 0.08	1.64 ± 0.07	NS
MAR_dist_	µm/D	1.98 ± 0.09	1.97 ± 0.03	NS		1.63 ± 0.10	1.62 ± 0.17	NS

Cylinders with geometric architecture contained significantly more titanium than those with trabecular architecture. The TitV/TV of the cylinders was similar between the two time series of analysis.

BV/TV did not differ between the two types of cylinders microarchitecture at 90 and 270 days. There was a slight increase in bone volume between 90 and 270 days but it did not reach significance.

**Figure 6.**
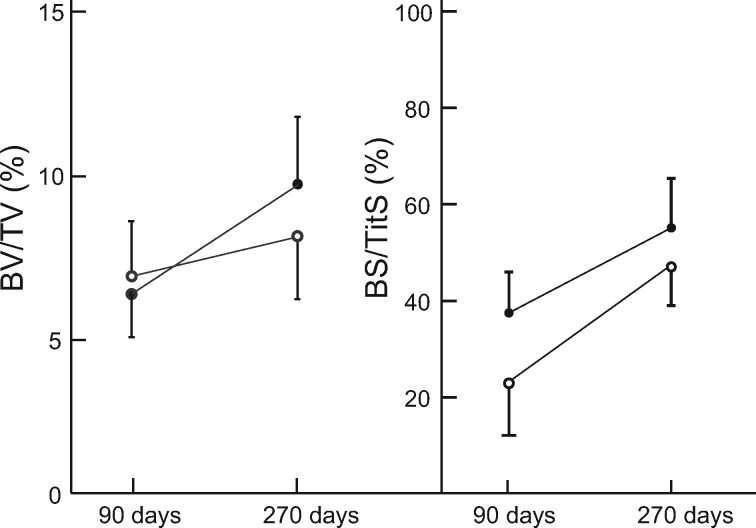
Evolution of histomorphometric parameters upon time. Opened circles represent cylinders with a trabecular microarchitecture; closed circles represent cylinders with a geometric microarchitecture

Similarly, the bone/titanium interface (BS/TitS) did not differ between the two types of cylinders microarchitecture and the increase was not significant between 90 and 270 days regardless of microarchitecture (Fig. 6).

The mineralization rate measured at the cylinder contact also did not differ between the two types of implants and was not altered at a 270 day after surgery. Similar results were observed for MAR_dist_ and surprisingly MAR_cont_ and MAR_dist_ did not differ at any time point.

## Discussion

Most of the studies concerning the osseointegration of biomaterials have been conducted on small laboratory animals [33, 34]. They are difficult to extrapolate to humans because the critical size defects are different [35, 36]. Filling a cavitary bone defect—which is similar to a critical size defect—in human orthopedic surgery remains a major problem [2, 37]. Studies in large animals such as ewes (which have large bones and a trabecular microarchitecture very similar to humans), are needed to qualitatively and quantitatively evaluate the bone response and the osseointegration process at the tissue level [4, 38].

In orthopedic surgery, several biomaterials are currently used to fill bone defects. These include bone (allogenic or xenogeneic) but calcium-orthophosphate ceramics have the advantage of safer bacterial and viral security [39–42]. Calcium phosphate ceramics exist as granules of different sizes that lead to an interconnected porosity (essential for osteoconduction and thus osseointegration) when placed in the grafted area [43–46]. Solid blocks of porous orthophosphate-based ceramics have also been developed in the past but the osseointegration results were poor due to the lack of interconnected porosity [47]. Large ceramic scaffolds with an interconnected porosity have been developed by industry but they do not have a sufficient mechanical strength in load-bearing bone [7, 9, 48].

To overcome the mechanical strength issues, metal-based biomaterials with an interconnected porosity have been developed by the industry [11]. Tantalum is known to be a biocompatible metal [15, 17] and Trabecular metal^®^ (Zimmer US), a tantalum-based metallic foam, is currently the only one available on the market for orthopedic surgery. It has an interconnected porosity and the microporous microarchitecture of the biomaterial (tetrakaidecahedron) would promote osseointegration.

In this study, porous titanium was chosen because this metal is known to promote osteoblast affinity [8, 49, 50]. Several studies have reported the use of titanium biomaterials with good tolerance and osseointegration properties [8, 24, 51, 52]. Furthermore, titanium has long been known to be an excellent biocompatible metal with good tolerance and excellent osseointegration properties and has been used in orthopedic and dental surgery for decades (see reviews in [17, 53]). Surgical specialties such as orthopedics, neurosurgery, dental and maxillofacial surgery very commonly use implants to replace bone. Since the loss of substance cannot always be managed by an autologous graft, biomaterials are needed to manage this type of problem. 3D printing by addictive manufacturing makes it possible to obtain implants of any shape, suitable for many conditions. Therefore, the industry can produce these biomaterials that have, in addition, an absolute viral or bacterial safety compared with auto, allo or xenomaterials.

In this study, we found that the titanium cylinders were osseointegrated into the femoral bone of ewes as evidenced by the presence of trabeculae anchored on the external surfaces of the cylinders. Only the use of large animals (goats or sheep) allows accurate studies of biomaterials that can be used in orthopedics, such as prostheses or biomaterials intended to fill large bone cavities in weight bearing areas. Large animal allow the graft of large biomaterial samples in bone defects of critical size, close to the lesions observed in humans [35, 36]. Sheep have a trabecular microarchitecture close to that of humans, which allows histomorphometric and histodynamic analyses to evaluate the osseointegration of biomaterials at the tissue level [4, 38]. Finally, biomaterials are most often evaluated in young laboratory animals with a vigorous osteoblastic function whereas their main indications remain for aged patients [54]. The use of older animals is rare in the literature because of the high costs of rearing and stabling for experimentation purposes [4, 54].

Both implants exhibited interconnected porosity that promotes osseointegration and differed only in their microarchitecture. Microarchitecture has been reported to be a determining factor in the bone tissue response leading to osseointegration [14, 55]. Bone cells are known to have varying affinities with surfaces with different topographic geometries [56, 57]. In this study, the geometric microarchitecture corresponded to that used for fusion cages used in spine surgery, so this microarchitecture is routinely used with satisfactory results on intervertebral fusion rates with good osseointegration [58, 59]. Implants with trabecular microarchitecture were prepared using bovine trabecular bone as a template in additive manufacturing. Our hypothesis was that such a scaffold mimicking bone microarchitecture would promote osseointegration more than a purely geometric microarchitecture [55]. However, the cylinder microarchitecture did not influence osseointegration because the amount of bone (BV/TV) and the BS/TitS were never different at any time. In both cases, bone extended from the surface to the center of the cylinder but most of it was in the first few millimeters. The bone modeling and remodeling was different from what is seen in dental surgery. Continuous remodeling has been repeatedly shown to occur on the surface of dental implants, resulting in increased BV/TV, BS/TitS and bone quality over time, associated with a reduced osteoblastic activity in an 18-month study [60, 61]. Mineralization rates were not significantly different over time with a slight trend toward ‘return to normal’. Our results appear to be very similar to those obtained with Trabecular metal^®^ tantalum foam in which osseointegration is limited to the most superficial areas of the implanted blocks [11, 62, 63]. Other authors have produced porous titanium materials with a lower porosity (23-32%) and a pore size of about 300 µm but they presented only *in vitro* results or indirect proofs of osseointegration in the rat without histological analysis [64, 65]. However, by additive printing, with a technology developed by Sirris, a reference center on this technique, it was not possible to obtain pores of size close to 300 µm without these being obstructed by the titanium powder or the porosity being interconnected. Here, large pores, already used for the fabrication of fusion cages in neurosurgery, were obtained using either a mathematical model or a replica of an aged bovine bone. The microarchitecture obtained is the same as that found in elderly human bones. In a recent paper, cylinders of porous titanium (6 mm in diameter) were also prepared by 3D printing with a mathematical model based on Voronoi 3D partition method [66]. In their study, the metal trabeculae were 200 µm in thickness without mentioning the pore size. Histological analysis revealed that the bone of the young sheep (2 years old) used in their study, had only partially colonized the titanium network by osteoconduction. In this series, a larger defect was created in aged ewes with the use of larger titanium cylinders.

Furthermore, it has been reported, in a series of 11 patients with these porous tantalum implants placed in the ankle that poor integration of the biomaterial results in non-union with bone. [10]. One explanation could be the absence of mechanical stress within the rigid implants whose pores create a protected volume disconnected from the stresses exerted on the graft. The absence of stress is deleterious to bone remodeling [67]. The phenomenon has been well described in orthopedic surgery and is known in hip arthroplasty as ‘stress-shielding’ [17, 68]. In the grafted cylinders in ewes, it can be assumed that integration occurs in the periphery where the mechanical stresses are sufficient to promote remodeling, whereas in the core of the cylinders, the stresses are too low and the bone cells do not perceive the signal. The absence of stress prevents remodeling.

In addition, another explanation for the lack of a statistically significant difference in the amount of bone grown in the titanium cylinders may be due to the positioning of the implant at the time of surgery. The cylinders were implanted parallel to the joint space. This difference could explain why the trabecular microarchitecture implants did not have the same mesh size as the neighboring bone of the ewes. On the contrary, the implants with geometric microarchitecture appeared much more regular. Finally, the question of the reproducibility of the surgical procedure for implanting the cylinders arises [4]. It can be assumed that depending on the orientation of the bone trabeculae in the operated bone, the cylinder is not placed parallel to the trabeculae and osseointegration is of poorer quality. In connection with the results of the fluid flow modeling, it is possible that implants mimicking the trabecular architecture require special placement to optimize osseointegration with the titanium trabeculae inserted parallel to the stress lines in the bone [23]. Cylinders with a geometrical architecture may not be subject to such a phenomenon because the fluid flow is the same whether the cylinder is vertical or horizontal. The CFD study shows that localized turbulent flow phenomena exist for implants with trabecular microarchitecture and that, conversely, implants with geometric microarchitecture exhibit laminar flow.

## Conclusion

Titanium implants allow long-term osseointegration over time. Although the microarchitecture of implant used does not seem to influence the quality of osseointegration, it may not be necessary to reproduce a microarchitecture close to natural bone whereas a geometrical architecture is easier to design and produce. 
